# Molecular cloning and characterization of a novel peptidase from *Trichinella spiralis* and protective immunity elicited by the peptidase in BALB/c mice

**DOI:** 10.1186/s13567-020-00838-1

**Published:** 2020-09-05

**Authors:** Jun Jun Lei, Yuan Yuan Hu, Fang Liu, Shu Wei Yan, Ruo Dan Liu, Shao Rong Long, Peng Jiang, Jing Cui, Zhong Quan Wang

**Affiliations:** Department of Parasitology, Medical College, Zhengzhou University, Zhengzhou, 450052 China

**Keywords:** *Trichinella spiralis*, peptidase, intrusion, intestinal epithelial cells (IECs), protective immunity

## Abstract

In our previous studies, a novel *T. spiralis* peptidase (TsP) was identified among the excretory/secretory (ES) proteins of *T. spiralis* intestinal infective larvae (IIL) and *T. spiralis* at the adult worm (AW) stage using immunoproteomics, but the biological function of TsP in the life cycle of *T. spiralis* is not clear. The objective of this study was to investigate the biological properties and functions of TsP in larval intrusion and protective immunity induced by immunization with rTsP. The complete TsP cDNA sequence was cloned and expressed. The results of RT-PCR, indirect immunofluorescence assay (IIFA) and western blotting revealed that TsP is a surface and secretory protein expressed in *T. spiralis* at different stages (muscle larvae, IIL, AWs and newborn larvae) that is principally localized at the epicuticle of the nematode. rTsP facilitated the larval intrusion of intestinal epithelial cells (IECs) and intestinal mucosa, whereas anti-rTsP antibodies suppressed larval intrusion; these facilitative and suppressive roles were dose-dependently related to rTsP or anti-rTsP antibodies. Immunization of mice with rTsP triggered an obvious humoral immune response (high levels of IgG, IgG1/IgG2a, and sIgA) and also elicited systemic (spleen) and intestinal local mucosal (mesenteric lymph node) cellular immune responses, as demonstrated by an evident increase in the cytokines IFN-γ and IL-4. Immunization of mice with rTsP reduced the numbers of intestinal adult worms by 38.6% and muscle larvae by 41.93%. These results demonstrate that TsP plays a vital role in the intrusion, development and survival of *T. spiralis* in hosts and is a promising candidate target molecule for anti-*Trichinella* vaccines.

## Introduction

*Trichinella spiralis* is an important foodborne nematode that parasitizes over 150 kinds of mammals worldwide [1]. Human *T. spiralis* infection is mainly caused by the ingestion of raw or undercooked meat infected with infectious encapsulated muscle larvae (ML). Pork- and pork-derived products from domestic pigs are major infectious sources of human trichinellosis in China [2, 3]. From 2004–2009, 12 trichinellosis outbreaks resulting from infected pork were documented in mainland China [4]. Because of the wide distribution of the natural animal hosts of *T. spiralis*, it is difficult to eliminate *T. spiralis* infection in food animals [5]. *Trichinella spiralis* infection causes enormous harm to human health and has become a serious threat to meat food safety [6, 7]. Hence, the need to develop vaccines to eradicate *Trichinella* infective larvae in food animals is imperative [8].

After infected meat is eaten, *T. spiralis* ML in muscle tissues are released from their collagen capsules with the help of gastric fluid digestion and activated to intestinal infective larvae (IIL) following exposure to the intestinal contents/bile [9]. These IIL larvae invade the intestinal mucosal columnar epithelium and develop to the adult worm (AW) stage after four moults. Females at the AW stage give birth to newborn larvae (NBL), which enter the blood system, penetrate the skeletal muscles and grow to become ML, completing the life cycle [10]. The intestinal epithelium is the primary native defence against *Trichinella* invasion and the principal site of interaction between IIL and the host [11, 12]. However, the mechanism of intestinal epithelium invasion by *Trichinella* worms has remained unclear. Characterization of molecules involved in *Trichinella* invasion will assist in elucidating the mechanism by which *T. spiralis* and its host interact and developing vaccines to inhibit *Trichinella* infection in animals [13, 14].

During *T. spiralis* infection, IIL excretory/secretory (ES) proteins are in contact with the host’s intestinal epithelial cells (IECs) and might have a major effect on IEC invasion [15, 16]. When IIL were cultivated with an IEC monolayer, the IIL penetrated the monolayer and produced some serine proteases that passed into the IECs [17, 18]. Moreover, diverse serine proteases have been identified among ES or surface proteins of *T. spiralis* worms at various stages using proteomics/immunoproteomics [19–22]. Additionally, the expression level of serine proteases in IIL was obviously higher than that in the ML [23]. These results suggest that serine proteases might participate in and promote invasion of the intestinal mucosal epithelium by IIL and intestinal infection [24]. Thus, serine proteases might be promising target molecules for a vaccine against intestinal *T. spiralis* infection [25–27].

All of the serine proteases and peptidase S1A subfamilies belong to the peptidase S1 family, the members of which have hydrolase and serine protease activities. Most members of the peptidase family are trypsin-like serine proteases based on their substrate specificity [28]. In previous studies, a novel *T. spiralis* peptidase from the S1A subfamily (TsP; GenBank: XM_003379300.1) was identified among the ES proteins of *T. spiralis* at the IIL and AW stages using immunoproteomics [29, 30], but its biological function in the life cycle of *T. spiralis* is not clear. The aims of the present study were to investigate the biological properties of TsP, to assess its roles in *T. spiralis* invasion and development and to evaluate protective immunity induced by immunization with rTsP.

## Materials and methods

### Worms and experimental animals

*Trichinella spiralis* worms (ISS534) were acquired from a domestic pig in central China [31] and maintained in our laboratory by serial passage in BALB/c mice. Four- to 6-week-old female BALB/c mice were purchased from the Henan Experimental Animal Center (Zhengzhou, China).

### Antigen preparation

ML were acquired by the artificial digestion of infected murine skeletal muscles at 40 days post-infection (dpi) [3]. IIL and AWs were collected from the small intestine of infected mice at 6 and 24 hpi and 3 and 6 dpi. Female AWs at 6 dpi were cultivated in RPMI-1640 medium with 10% foetal bovine serum (FBS; Gibco, Thermo Fisher Scientific, Waltham, MA, USA) at 37 °C in 5% CO_2_ for 72 h, and the NBL were collected as previously described [32, 33]. Soluble crude somatic proteins and ES proteins from *T. spiralis* at diverse stages (ML, IIL, AWs and NBL) were prepared as reported before [34].

### Bioinformatics analysis of TsP

The full-length TsP gene cDNA sequence was retrieved from GenBank (GenBank: XP_003379348.1). The physicochemical characteristics of TsP were predicted through bioanalytical software and websites. The presence of signal peptides in TsP and subcellular localization of TsP were analysed as described previously [35]. The tertiary structure of TsP was predicted with PyMOL software (DeLano Scientific LLC, San Carols, CA, USA), and its functional sites were analysed using CN3D software (NCBI, Bethesda, MD, USA) [14]. The amino acid sequence of the TsP gene was compared with that of peptidase from other organisms with Clustal X; the GenBank accession numbers of the peptidases from other organisms used for this comparison were as follows: *Trichinella nelsoni* (KRX23647.1)*, T. nativa* (KRZ55862.1), *T. britovi* (KRX45325.1), *Trichinella* sp. T9 (KRX64877.1), *T. patagoniensis* (KRY21930.1), *Trichinella* sp. T6 (KRX75765.1), *T. murrelli* (KRX45325.1), *T. papuae* (KRZ76774.1), *Mus musculus* (AAA50168.1) and *Homo sapiens* (CAE48420.1). Phylogenetic analysis was performed using MEGA 7.0 based on the neighbour-joining (NJ) method as reported before [36].

### Cloning, expression and identification of TsP

Total RNA was extracted from the ML using TRIzol (Invitrogen, Carlsbad, CA, USA). The full-length cDNA sequence of the TsP gene was amplified by PCR using the following specific primers carrying the restriction enzyme sites BamHI and HindIII **(bold**) (5′-AC**GGATCC**ATGGAAATTTATCAGCTGAG-3′ and 5′-GCG**AAGCTT**TCAGCTTGGCAGATATTTAT-3′). The PCR product was cloned into the expression vector pQE-80L, and the recombinant pQE-80L/TsP plasmid was transformed into *Escherichia coli* BL21 (DE3) (Novagen, La Jolla, CA, USA). rTsP expression was induced by incubation with 0.8 mM IPTG at 30 °C for 6 h [37], and rTsP was purified with Ni–NTA Sefinose resin (Sangon Biotech Co., Shanghai, China) [38]. The rTsP protein concentration was ascertained, and the rTsP was analysed by SDS-PAGE and western blotting as previously described [22].

### Immunization of mice and ELISA-mediated determination of anti-rTsP antibodies

Ninety mice were randomly divided into 3 groups (30 mice per group). Each mouse was subcutaneously injected with 20 µg of rTsP emulsified with the adjuvant ISA 201 (Seppic, Paris, France) and boosted three times with rTsP with ISA 201 at a 2-week interval. Control mice received only ISA 201 or PBS alone at the same times mice in the experimental group were vaccinated [39]. 100 μL of tail blood was collected from each mouse at weeks 0, 2, 4, 6 and 8 after vaccination, and serum samples were isolated and stored at − 80 °C until use [40].

Specific anti-rTsP IgG and IgG1/IgG2a antibodies in all vaccinated mice were measured by conventional ELISA with rTsP as the coating antigen [41]. In brief, plates were coated with 2 μg/mL rTsP and incubated at 4 °C overnight. After washing with PBST, the plates were blocked with 5% skimmed milk at 37 °C for 2 h. After washing with PBST, the plates were incubated at 37 °C for 2 h with 1:100 dilutions of murine immune sera, followed by incubation with HRP-labelled anti-mouse IgG (IgG1/IgG2a; 1:5000 dilution; Sigma-Aldrich, St. Louis, MO, USA) at 37 °C for 1 h. The signal was developed using OPD (Sigma-Aldrich) plus H_2_O_2_, and the reaction was terminated by the addition of 2 M H_2_SO_4._ The absorbance (optical density, OD) at 492 nm was assayed using a microplate reader (Tecan, Schweiz, Switzerland) [34, 38].

### RT-PCR analysis of TsP transcription in *T. spiralis* at various stages

Total RNA was extracted from ML, IIL, 3-day-old AWs, 6-day-old AWs and NBL using TRIzol reagent (Invitrogen). RT-PCR was performed to assess the TsP transcription levels in *T. spiralis* at various stages as previously reported [27]. An internal control gene (GAPDH) from *T. spiralis* (GenBank: AF452239) was also amplified. PBS was used as a negative control in all PCR experiments.

### Indirect immunofluorescence assay (IIFA)

Fresh intact *T. spiralis* at various stages (ML, IIL, AWs and NBL) were fixed in cold acetone for 20 min. Then, the ML, IIL and AWs were embedded in paraffin, and 3-µm-thick cross-sections of the worms were cut with a microtome. The expression and localization of native TsP in *T. spiralis* at various stages were observed using IIFA [42, 43]. Briefly, whole worms and worm cross-sections were blocked with 5% goat serum and then probed using different sera (1:10; anti-rTsP serum, infection serum or pre-immune serum) at 37 °C for 2 h. Following washes with PBS, worms and worm cross-sections were incubated with FITC-anti-mouse IgG conjugate (1:100; Santa Cruz, Dallas, Texas, USA). After washing with PBS again, the complete worms and worm cross-sections were observed under a fluorescence microscope (Olympus, Tokyo, Japan) [44, 45].

### IIFA of the binding of rTsP with the enteral epithelium

The small intestine, liver and lung were collected from normal mice, and the tissues were fixed in 4% formaldehyde. Tissue section (3 μm) were cut with a microtome. IIFA was conducted as described previously [46]. Briefly, the sections were incubated with 20 µg rTsP for 2 h at 37 °C. After blocking and washing, the sections were probed with 1:10 dilutions of anti-rTsP serum, infection serum or pre-immune serum for 2 h at 37 °C and then stained using FITC-labelled anti-mouse IgG conjugate diluted 1:100 (Santa Cruz). The sections were re-stained for 5 min with 4′,6-diamidino-2-phenylindole (DAPI) [47]. Finally, the sections were observed under a fluorescence microscope (Olympus) [48].

### Detection of rTsP and IEC binding by IIFA

The binding of rTsP and IECs and the cellular location of rTsP were assessed by IIFA and confocal microscopy [11, 49]. IECs in DMEM were cultured on a coverslip in a 6-well plate [50]. The IEC monolayer was pre-incubated with 20 µg/mL rTsP at 37 °C for 2 h and blocked with 10% goat serum for 1 h. After washing with PBS, the monolayer was incubated with anti-rTsP serum (1:10) at 37 °C for 1 h. After washing again, the cells were stained at 37 °C for 1 h using FITC-conjugated anti-mouse IgG (1:100, Santa Cruz), and the cell nuclei were re-stained with DAPI for 5 min and observed with fluorescence microscopy (Olympus). Finally, the cellular localization of TsP in IECs was ascertained by confocal microscopy [27].

### Far-western blot identification of the binding between rTsP and IEC proteins

The binding of rTsP with IEC proteins was examined by far-western blotting. Briefly, IEC lysates were separated by SDS-PAGE with a 12% separating gel. The proteins were transferred onto a nitrocellulose membrane, which was cut into strips, blocked, incubated with rTsP and probed with anti-rTsP serum. After washing with PBST, the strips were incubated with HRP-conjugated anti-mouse IgG (1:5000, Sigma-Aldrich). After washing, the signal was developed using 3,3′-diaminobenzidine tetrahydrochloride (DAB; Sigma-Aldrich,) [51].

### In vitro larval invasion of IECs

To investigate the role of TsP in *T. spiralis* invasion of the intestinal epithelium, an in vitro larval invasion test was conducted as previously described [52, 53]. Briefly, ML were first activated into IIL by using 5% swine bile at 37 °C for 2 h, and 100 IIL were added to an IEC monolayer [54]. The culture medium had been supplemented with serial diluted rTsP protein (2.5, 5.0, 7.5, 12.5 and 15.0 μg/mL) or serial dilutions (1:100–1:1000) of anti-rTsP serum, infection serum or pre-immune serum. After culture at 37 °C for 2 h, larval invasion of the monolayer was examined under a microscope. The IIL that penetrated the IEC monolayer and migrated were assessed as invaded larvae, whereas the larvae that remained on the IEC surface and exhibited a spiral coil-like shape were considered non-penetrated worms [43]. Each test was performed in triplicate.

### rTsP-mediated promotion and anti-rTsP serum-mediated inhibition of larval invasion of the excised intestine

The role of rTsP in larval invasion of the enteral mucosa was also evaluated using the excised mouse small intestine as previously reported [49]. Two hundred IIL were first mixed with anti-rTsP serum diluted 1:100–1:1000 and 0–15 μg/mL rTsP or BSA. The intestines were collected from normal mice, washed with sterilized Tyrode’s solution, and then cut into 2 cm-long segments. The two enteral ends were ligated to form an intestinal pouch, and the IIL were injected into the enteral lumen and kept in sterilized Tyrode’s solution for 2 h at 37 °C. Each test was performed in triplicate, and the numbers of larvae that retained in the enteral lumen were assessed as non-invaded worms.

### Assessment of enteral total IgA and TsP-specific sIgA levels

To ascertain total and TsP-specific secretory IgA (sIgA) levels in the intestinal fluid, intestinal eluent was prepared as reported before [55, 56]. Briefly, a 20-cm-long intestinal segment was cut, and the enteral interior was washed three times using 1 mL of cold PBS with 1% protease inhibitor (Sangon Biotech, Shanghai, China). The eluents were collected and centrifuged at 5000 × *g* for 5 min, and the supernatants were harvested [26]. Total intestinal sIgA levels were determined using sandwich ELISA as previously described [44]. TsP-specific sIgA was detected by ELISA using 2 μg/ml soluble antigens from ML or rTsP as coating antigens [57]. The signal was developed with OPD, and the absorbance at 492 nm was assayed as described previously [40].

### ELISA to determine the TsP-specific cytokine response

To assess TsP-specific cellular immune responses, five immunized mice from each group were killed at weeks 0 and 8 post-immunization. Murine spleens and mesenteric lymph nodes (MLNs) were harvested and homogenized in complete RPMI-1640 medium (Gibco, Auckland, New Zealand), the pellets after centrifugation at 1500 × *g* for 10 min were collected, and the cells were isolated as previously described [56, 58]. The cell density was adjusted to 2 × 10^6^cells/mL in RPMI-1640 medium with 5% FBS, penicillin (100 U/mL) and streptomycin (100 μg/mL). The cells were stimulated using 4 μg/mL rTsP for 72 h at 37 °C and 5% CO_2_. The supernatant was collected, and the levels of two cytokines (IFN-γ and IL-4) were determined with sandwich ELISA [40]. Cytokine concentrations are shown as picograms per millilitre (pg/ml).

### Larval challenge and evaluation of immune protection

To evaluate the immune protection induced by rTsP immunization, each immunized mouse was challenged using 300 *T. spiralis* ML at 2 weeks after the last immunization. The AWs were collected from the small intestines of ten immunized mice from each group at 6 dpi. The ML were obtained by the artificial digestion of skeletal muscle from the remaining ten immunized mice from each group at 30 dpi. The immune-protective efficacy of rTsP immunization was evaluated as the reduction in intestinal AWs and muscle larvae per gram (LPG) of immunized mice and compared to the number of intestinal AWs and muscle LPG in the PBS group [59, 60].

### Histological examination of intestines and muscles from infected mice

On 6 and 30 dpi, the gut and masseter muscles were cut from infected mice, fixed in 4% formalin for 24 h and embedded in paraffin. Tissue sections (3-μm thick) were prepared, deparaffinized and stained using haematoxylin and eosin (HE) staining. The sections were examined under microscopy, and the inflammatory cells (eosinophils, neutrophils and lymphocytes) per field (×200) were counted as previously described [56, 61].

### Statistical analysis

All the data were statistically analysed with SPSS for Windows, version 20.0. The data are shown as the mean ± standard deviation (SD). Differences among different groups were analysed by a Chi square test or one-way ANOVA. Correlation analysis was used to assess the relationship between larval invasion and the dilution of rTsP/anti-rTsP serum. Differences for which *P* < 0.05 were considered statistically significant differences.

## Results

### Bioinformatics analysis of TsP

The full-length TsP cDNA sequence is 780 bp long and encodes 259 aa, and the encoded protein has a molecular weight (MW) of 28.7 kDa and isoelectric point (pI) of 8.32. TsP has no signal peptide, and TMHMM prediction showed that TsP has no transmembrane domain but does have a functional domain named Tryp_SPc located from aa 21–247. The results of homology comparison of the TsP amino acid sequence with the amino acid sequences of peptidases from other *Trichinella* species or genotypes are shown in Additional file 1. The amino acid sequence of TsP exhibited identities of 86.67, 83.40, 83.02, 82.26, 81.51, 80.90 and 80.38% with peptidases from 7 encapsulated *Trichinella* species (*T. nelsoni, T. nativa, T. britovi*, T9, *T. patagoniensis*, T6 and *T. murrelli*) and an identity of 74.25% with the peptidase from a non-encapsulated *Trichinella* species (*T. papuae*).

**Figure 1:**
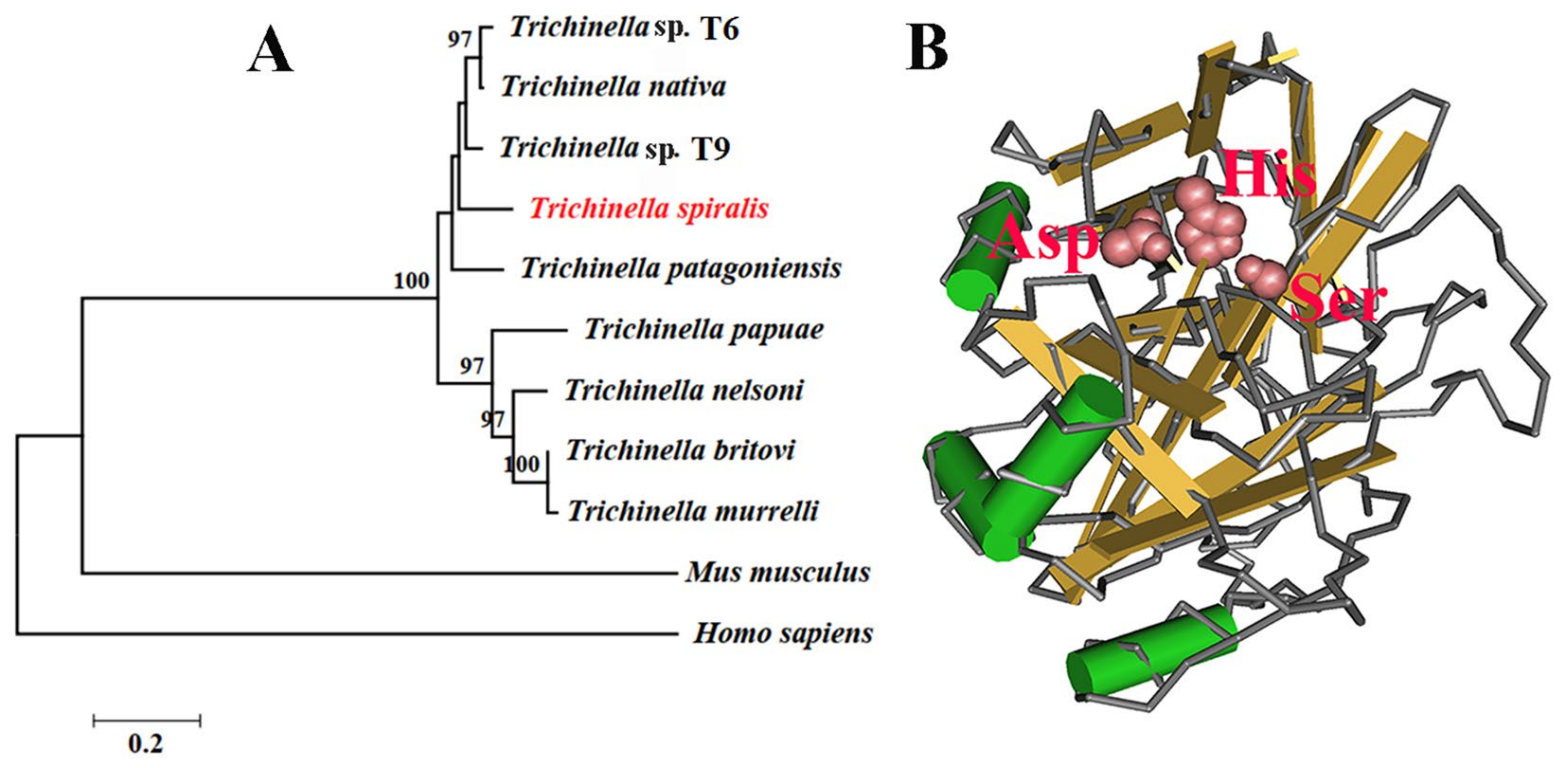
**Phylogenetic tree consisting of peptidases from 11 organisms determined with the NJ method (A) and the predicted 3-dimensional structure of the TsP protein (B).** Three serine protease-specific active site residues (His, Asp and Ser) in TsP are coloured red.

The result of phylogenetic analysis of TsP and peptidases from other *Trichinella* species or genotypes is shown in Figure 1A. The phylogenetic tree strongly supports the presence of a monophyletic group consisting of the genus *Trichinella*. Within the genus *Trichinella*, *T. spiralis* has a closer evolutionary relationship with the encapsulated *T. nativa*, *Trichinella* sp. T6 and T9 genotypes, as shown based on the phylogenetic analysis of their peptidases. Structure prediction showed that TsP has 4 α-helices, 16 β-strands, and a domain (between aa 21 and 247) with trypsin-like serine protease activity and an active site carrying the classic catalytic triad. In the three-dimensional model, the catalytic triad serine–histidine–aspartate forms a functional domain with substrate-binding sites (Figure 1B).

### SDS-PAGE and western blotting identification of the rTsP protein

**Figure 2:**
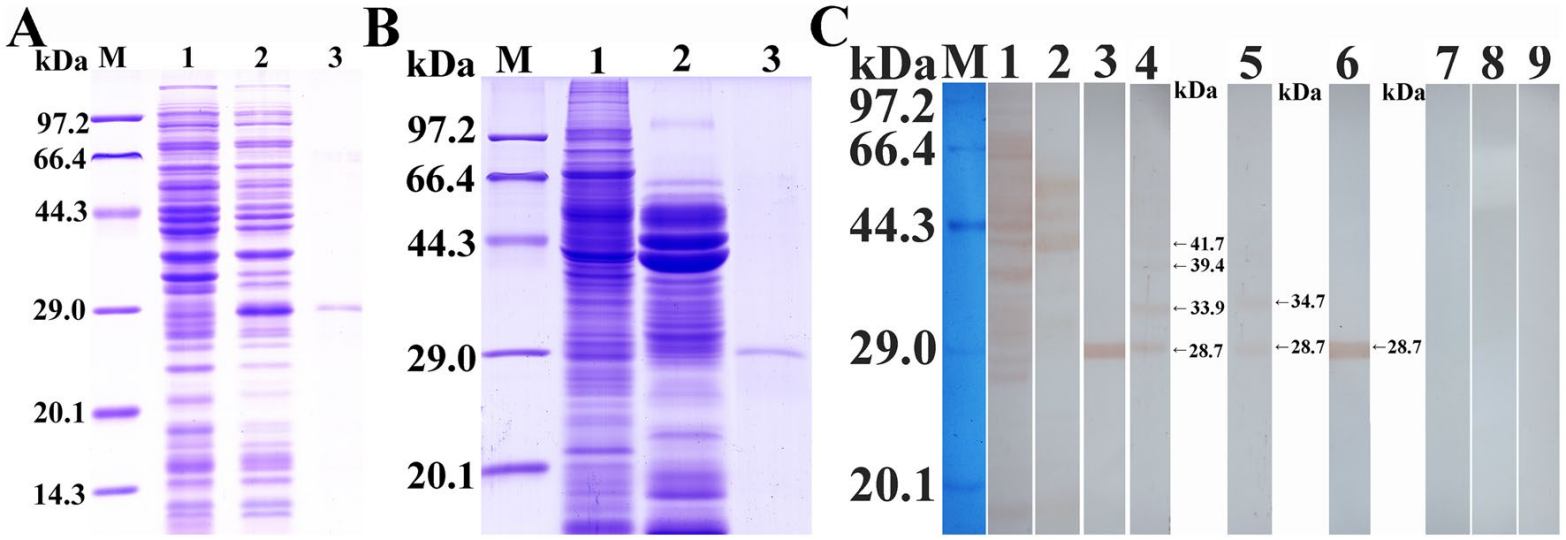
**SDS-PAGE and western blot analysis of rTsP. A** SDS-PAGE analysis of lysates of BL21 bacteria transfected with pQE-80L/TsP before and after induction. Lane M: protein marker; lane 1: lysate of BL21 bacteria transfected with pQE-80L/TsP prior to induction; lane 2: lysate of BL21 bacteria transfected with pQE-80L/TsP following induction; lane 3: purified rTsP. **B** SDS-PAGE analysis of rTsP. Lane M: protein marker; lane 1: ML soluble proteins; lane 2: ML ES proteins; lane 3: purified rTsP. **C** Western blot analysis of rTsP antigenicity. ML soluble proteins (lane 1), ML ES proteins (lane 2) and rTsP (lane 3) were identified by *T. spiralis*-infected sera. Native TsP among ML soluble proteins (lane 4) and ES proteins (lane 5), and rTsP (lane 6) was recognized by anti-rTsP serum. ML soluble proteins (lane 7), ES proteins (lane 8), and rTsP (lane 9) were not recognized by normal murine serum.

SDS-PAGE showed that recombinant BL21 bacteria carrying PQE-80L/TsP expressed a fusion protein 28.7 kDa in size. After purification, rTsP was present as a clear, single protein band (Figure 2A, B). The MW (28.7 kDa) of rTsP determined by SDS-PAGE was identical to its predicted MW (28.7 kDa).

Western blotting showed that soluble and ES proteins and rTsP from ML were recognized by *T. spiralis*-infected serum and anti-rTsP serum but not normal murine serum. Native TsP constructs with MW from 28.7–41.7 kDa were identified among the soluble and ES proteins of *T. spiralis* ML by anti-rTsP serum (Figure 2C), demonstrating that TsP was expressed among ML ES proteins and that TsP is a ML secretory protein.

### TsP mRNA transcription in *T. spiralis* at various stages

**Figure 3:**
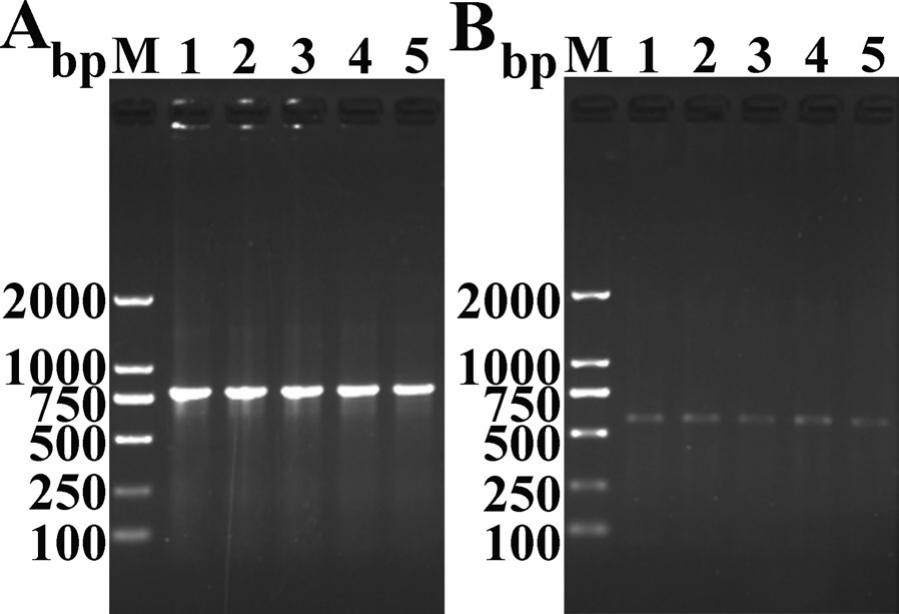
**RT-PCR analysis of transcription of the TsP (A) and GAPDH (B) genes in** ***T. spiralis*** **at diverse stages.** Lane M: DNA marker; lane 1: ML; lane 2: IIL; lane 3: 3-day-old AWs; lane 4: 6-day-old AWs; lane 5: NBL.

The RT-PCR results showed transcription of the TsP gene (780 bp) in *T. spiralis* at various stages of its life cycle (e.g., ML, IIL, 3- and 6-day-old AWs, and NBL) (Figure 3A), and the housekeeping gene (GAPDH) was amplified from *T. spiralis* at all stages and showed the expected size (570 bp) (Figure 3B).

### Expression and location of native TsP in *T. spiralis* at various stages determined by IIFA

**Figure 4:**
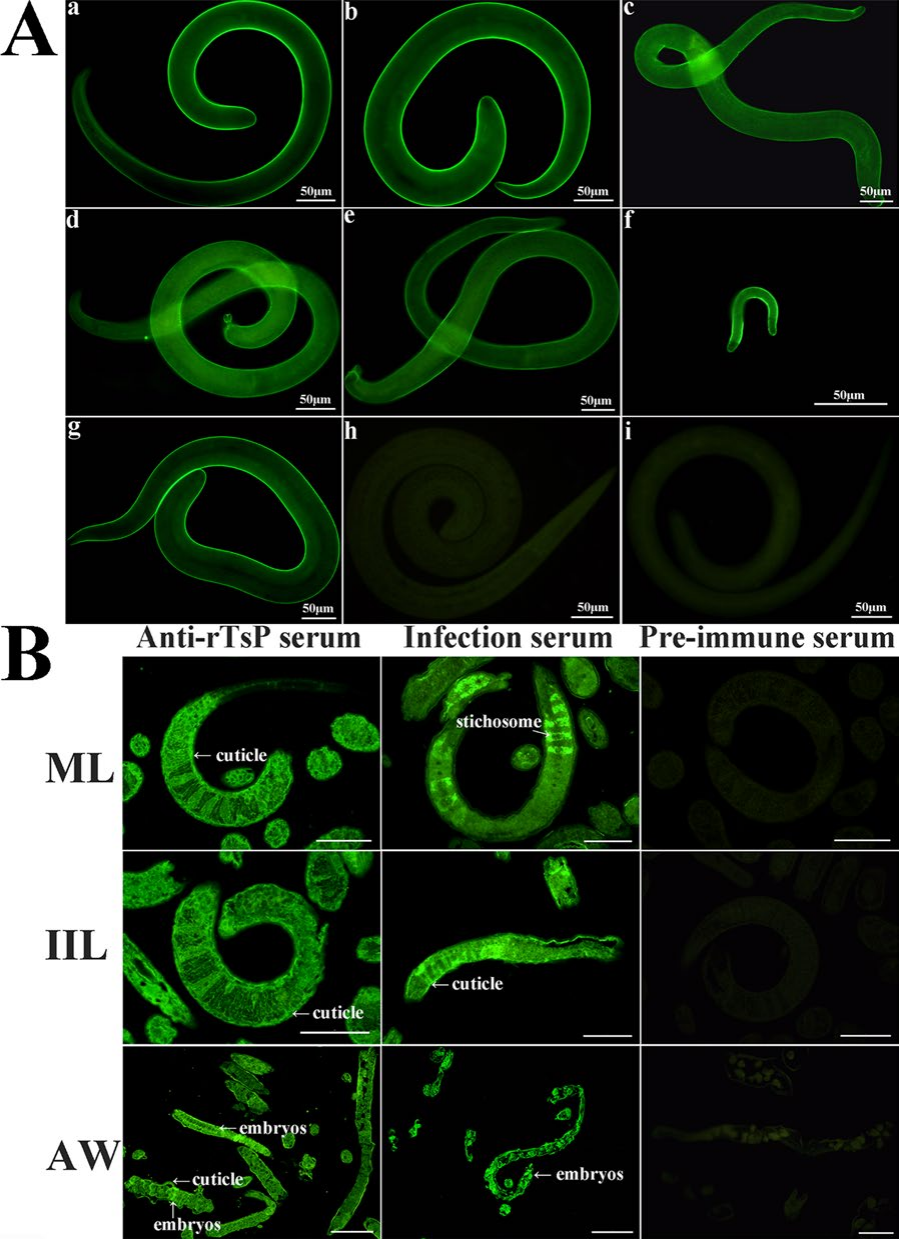
**Expression and immunolocalization of TsP in** ***T. spiralis*** **at various stages by IIFA using anti-rTsP serum. A** Expression of TsP at the epicuticle of *T. spiralis* at various stages was determined by IIFA. Intact whole worms were probed by anti-rTsP serum, and immunostaining was observed on the cuticle of worms at various stages. **a** ML; **b** 6 h IIL; **c** 24-h IIL; **d** 3-day-old AWs; **e** 6-day-old AWs; **f** NBL. **g** ML probed using *T. spiralis*-infected serum were used as a positive control. ML probed with pre-immune serum (**h**) and PBS (**i**) were used as a negative control. Scale bars = 50 μm. **B** Immunolocalization of TsP in worm cross-sections from *T. spiralis* at various stages was determined by IIFA using anti-rTsP serum. Immunostaining was observed at the cuticle of ML, IIL, and intrauterine embryos of female AWs. No immunostaining on worm cross-sections was observed when pre-immune serum was used as a negative control. ML and IIL scale bars: 50 μm. AW scale bars: 100 μm.

The results of IIFA of complete worms revealed upon anti-rTsP serum application, bright green fluorescent staining was found on the epicuticle of *T. spiralis* at various stages (ML, 6- and 24-h-old IIL, 3- and 6-day-old AWs and NBL) (Figure 4A). When the worm cross-sections were probed by anti-rTsP serum, immunostaining was located at the cuticle and intrauterine embryos of the parasite (Figure 4B). No immunostaining was observed using pre-immune serum.

### Specific binding of rTsP with the enteral epithelium assessed by IIFA

**Figure 5:**
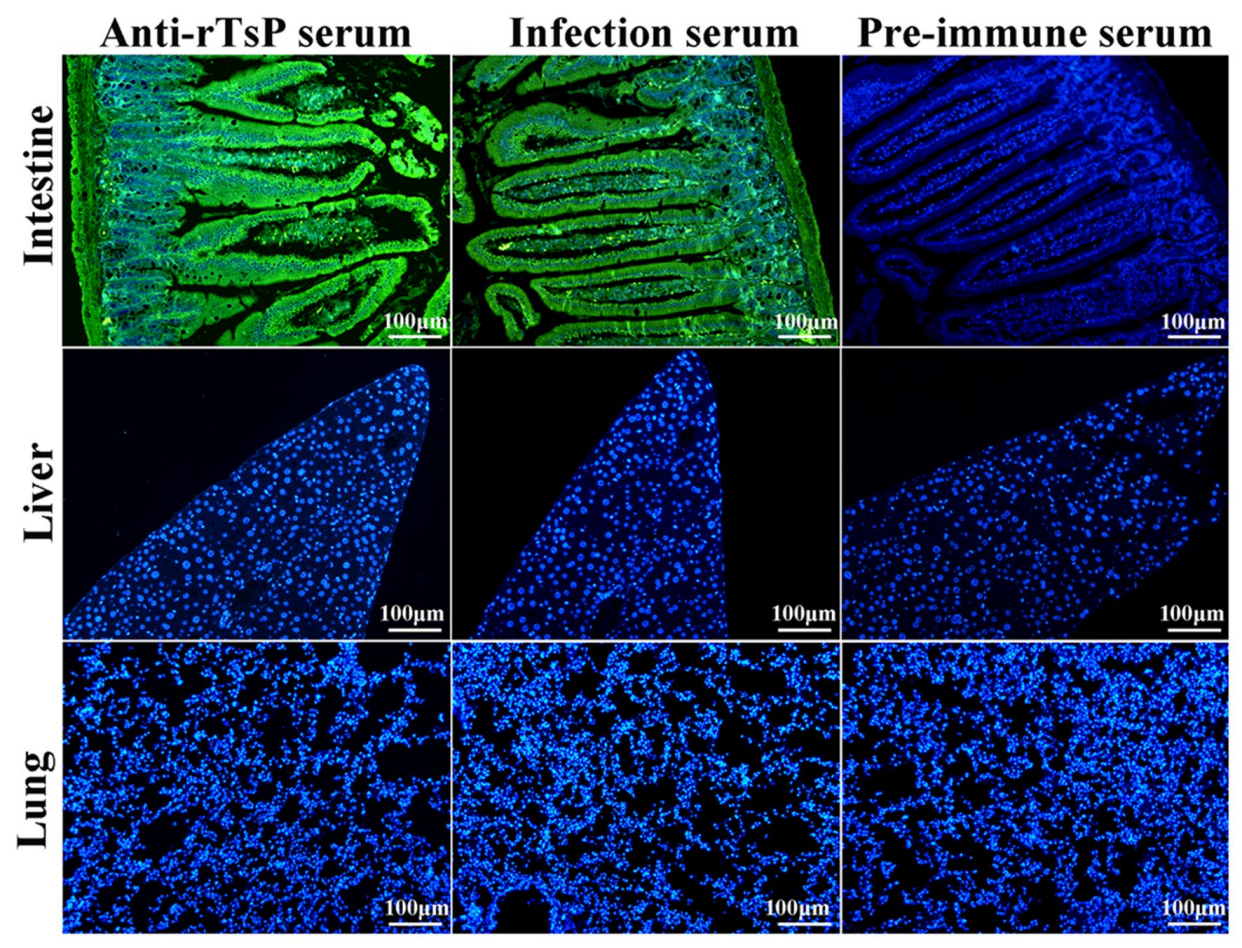
**IIFA analysis of the binding of rTsP to the normal murine enteral epithelium (100×).** Tissue sections from the normal murine intestine, liver and lung were first incubated with rTsP at 37 °C for 2 h. After washing, the sections were probed at 37 °C for 1 h with anti-TsP serum, infection serum or pre-immune serum and then stained using anti-mouse IgG-FITC conjugate. DAPI was used to stain the cell nuclei blue. The sections were observed under fluorescence microscopy. Scale bars: 100 μm.

The results of IIFA using enteral, liver and lung sections showed immunostaining of the enteral epithelium after its incubation with rTsP, anti-rTsP serum and infection serum (Figure 5). However, no immunostaining was observed with pre-immune serum. The liver and lung tissue sections incubated with rTsP did not show any detectable staining by anti-rTsP serum or infection serum.

### Binding of rTsP with IECs and the cellular localization of rTsP

**Figure 6:**
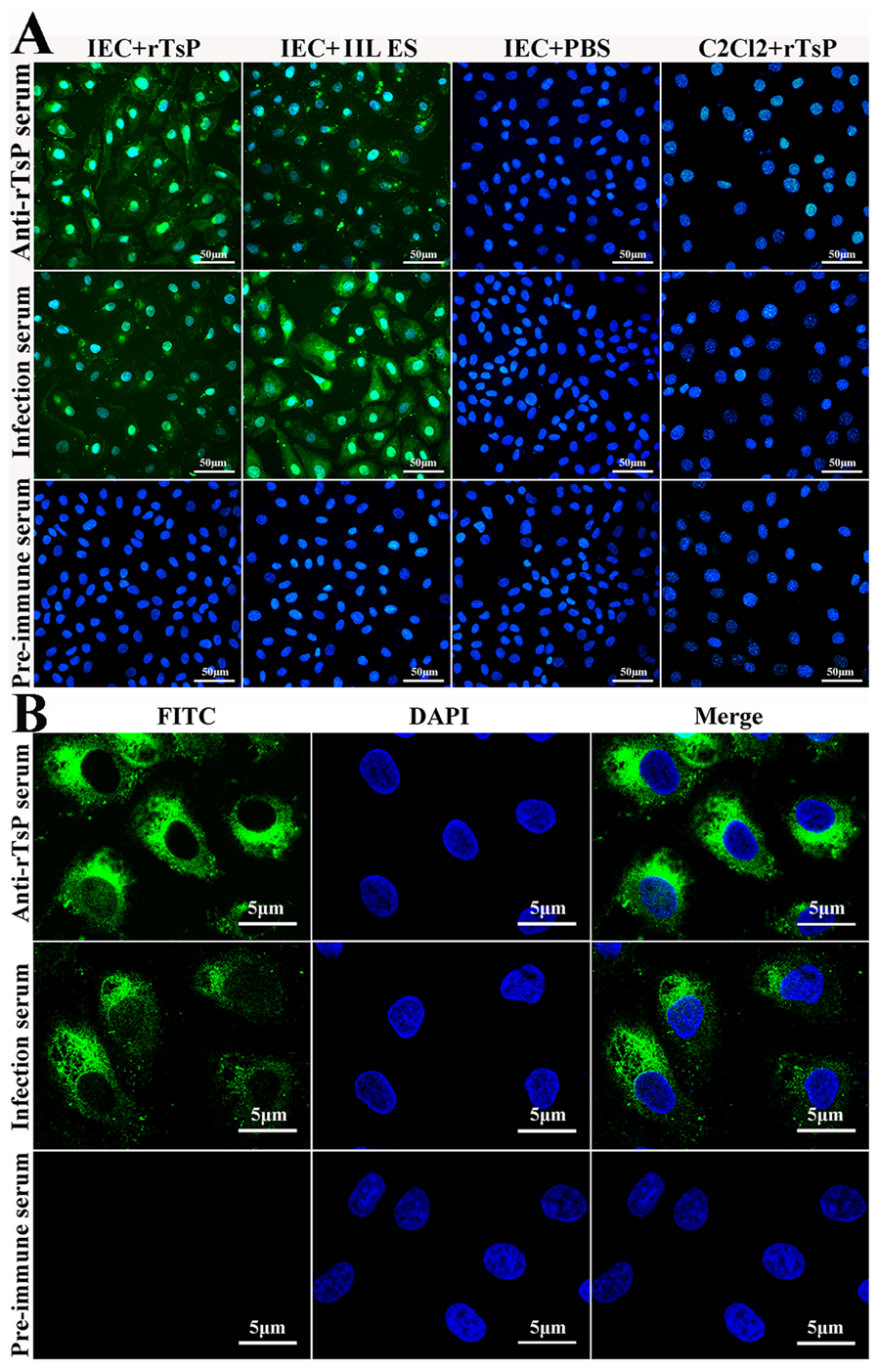
**The specific binding of rTsP with IECs and the cellular localization of rTsP. A** IIFA was used to identify the specific binding of rTsP with IECs. IECs or C2C12 cells were pre-incubated with rTsP, IIL ES antigens or PBS. After blocking and washing, the IECs and C2C12 cells were incubated with anti-rTsP serum, infection serum or pre-immune serum, followed by incubation with FITC-conjugated anti-mouse IgG. Cell nuclei were re-dyed blue using DAPI. Scale bars: 50 μm. **B** The cellular localization of rTsP in IECs was determined by confocal microscopy. The IECs were first pre-incubated with rTsP; probed with anti-rTsP serum, infection serum or pre-immune serum; and finally stained using FITC-conjugated anti-mouse IgG. Scale bars: 5 μm.

The results of IIFA revealed that after the IECs were pre-incubated with rTsP and IIL ES antigens, immunostaining was observed on the surface of IECs probed by anti-rTsP serum and infection serum but not pre-immune serum. The IECs pre-incubated with PBS alone did not show immunostaining. No immunostaining for C2C12 was observed when C2C12 cells pre-incubated with rTsP were probed with anti-rTsP serum or infection serum (Figure 6A). The results of confocal microscopy indicated that immunostaining was primarily located in the IEC cytoplasm (Figure 6B), demonstrating the specific binding of rTsP with IECs and that the rTsP-binding site is principally localized in the cytoplasm.

### The binding of rTsP and IEC proteins determined by far-western blotting

**Figure 7:**
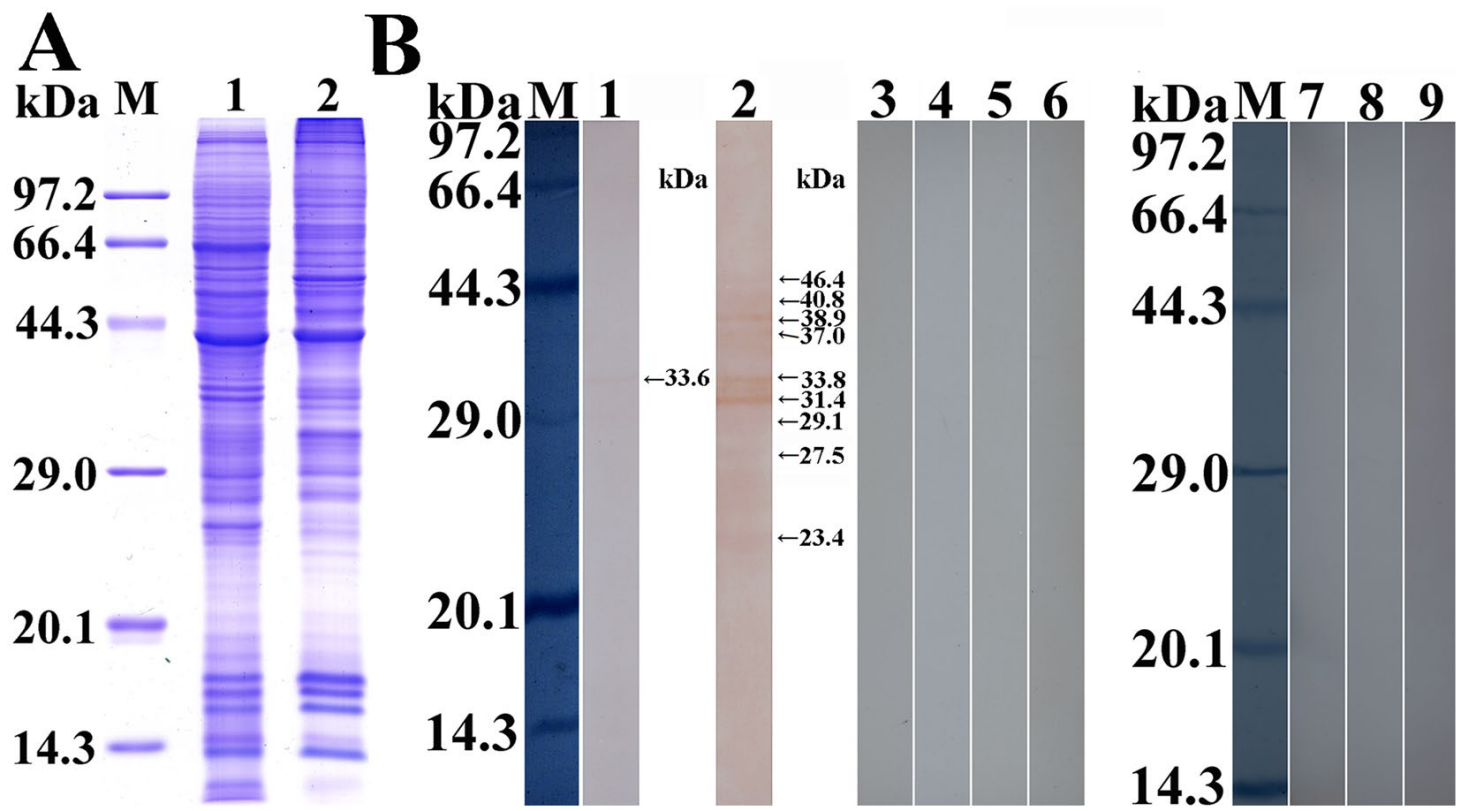
**Far-western blotting of rTsP binding with IEC proteins. A** SDS-PAGE analysis of IEC (lane 1) and C2C12 cell (lane 2) lysates. **B** The specific binding between rTsP and IEC proteins was assessed by far-western blotting. A membrane carrying IEC proteins (lanes 1–6) was incubated with rTsP (lanes 1–3) or BSA (lanes 4–6). Specific bands of rTsP bound with IECs were identified by the application of infection serum (lane 1) and anti-rTsP serum (lane 2) but not pre-immune serum (lane 3). No binding between BSA and IEC proteins was detected with infection serum (lane 4), anti-rTsP serum (lane 5), or pre-immune serum (lane 6). No binding of rTsP with C2C12 cells was detectable using infection serum (lane 7), anti-rTsP serum (lane 8), or pre-immune serum (lane 9).

After IEC proteins were incubated with rTsP, one band with a MW of 33.6 kDa was recognized by infection serum, and 9 bands with MWs from 23.4 to 46.4 kDa were recognized by anti-rTsP serum. No proteins in IECs pre-incubated with rTsP were identified by pre-immune serum, and no immunostaining for the C2C12 protein was detected with anti-rTsP serum or infection serum after pre-incubation with rTsP (Figure 7). These results indicated the specific binding of TsP and IEC proteins.

### rTsP-mediated facilitation or anti-rTsP serum-mediated inhibition of the larval invasion of IECs

**Figure 8:**
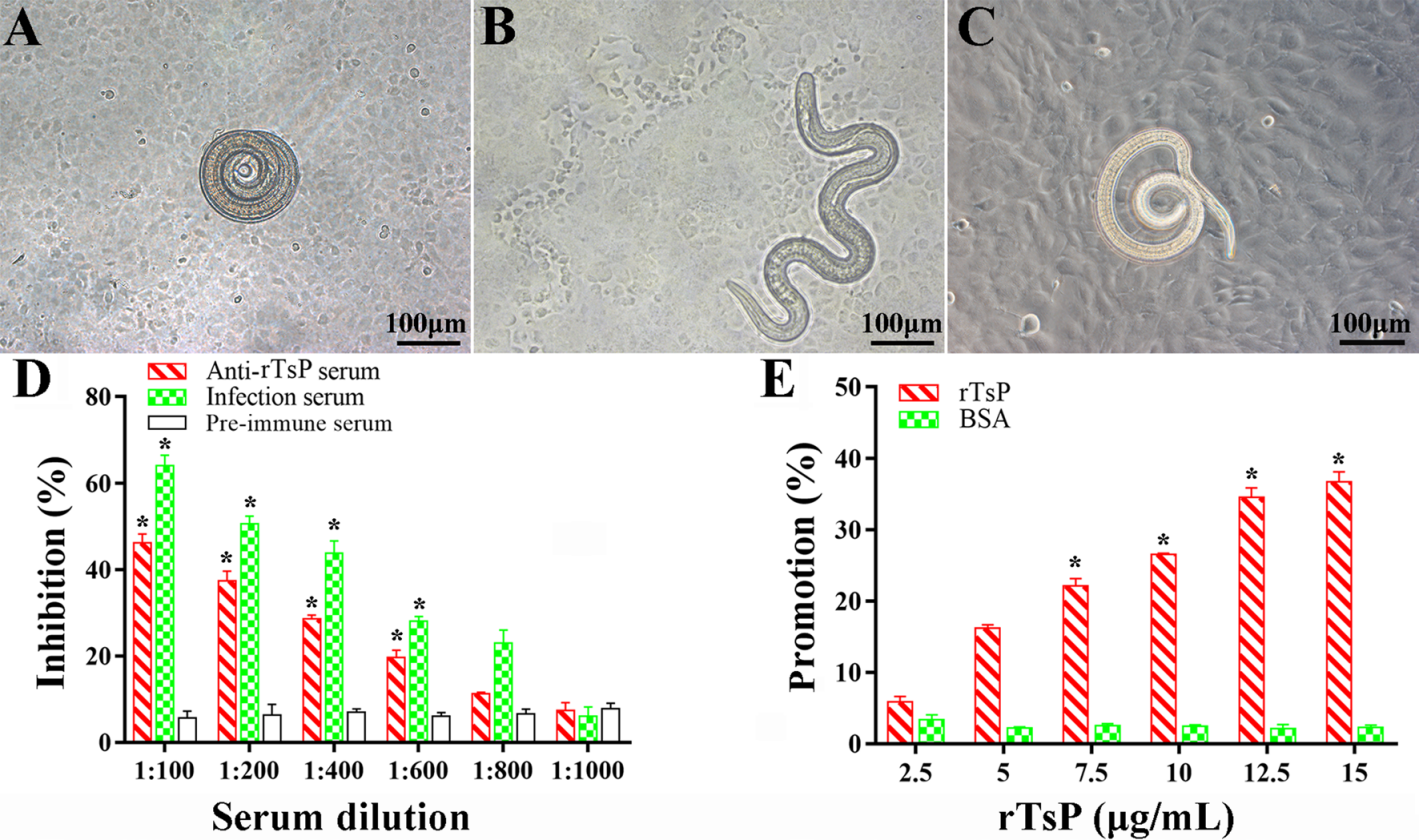
**rTsP-mediated promotion and anti-rTsP antibody-mediated inhibition of the larval invasion of IECs.** *Trichinella spiralis* ML were first activated into IIL using 5% swine bile for 2 h at 37 °C, after which the IIL were added to an IEC monolayer. The IIL that invaded the IECs were examined under microscopy after culture for 2 h. **A** Non-invaded IIL coiled on the IEC surface. **B** Larvae that invaded the IEC monolayer were mobile and migratory. **C** Non-invaded IIL coiled on the surface of C2C12 cells, which were insensitive to larval invasion and utilized as negative control cells. **D** Inhibitory effect of anti-rTsP serum on IIL invasion of IECs. **E** rTsP promoted the IIL invasion of IECs. *P < 0.01 compared to pre-immune serum or BSA. Scale bars: 100 μm.

After cultivation with the IEC monolayer for 2 h, IIL that invaded the monolayer were examined (Figure 8A–C). When serial dilutions of anti-rTsP serum were used to supplement the culture medium and incubated with IECs for 2 h, anti-rTsP serum (1:100–1:600) obviously inhibited larval invasion compared to that in the pre-immune serum group (*P *< 0.01). The suppressive effect of anti-rTsP antibodies was dose-dependent (*r *= 0.906), and larval invasion declined with increasing serum dilutions (*F *= 254.071, *P *< 0.0001) (Figure 8D). When the medium was supplemented with rTsP and cultivated with IIL for 2 h, rTsP clearly promoted IEC invasion by IIL. This effect was dependent on the dose of rTsP (*r *= 0.985), and IEC invasion tended to increase with increasing rTsP concentration (*F *= 432.176, *P *< 0.0001); however, BSA did not play a role in promoting larval invasion (Figure 8E).

### rTsP-mediated promotion and anti-rTsP serum-mediated suppression of larval invasion of the enteral mucosa

**Figure 9:**
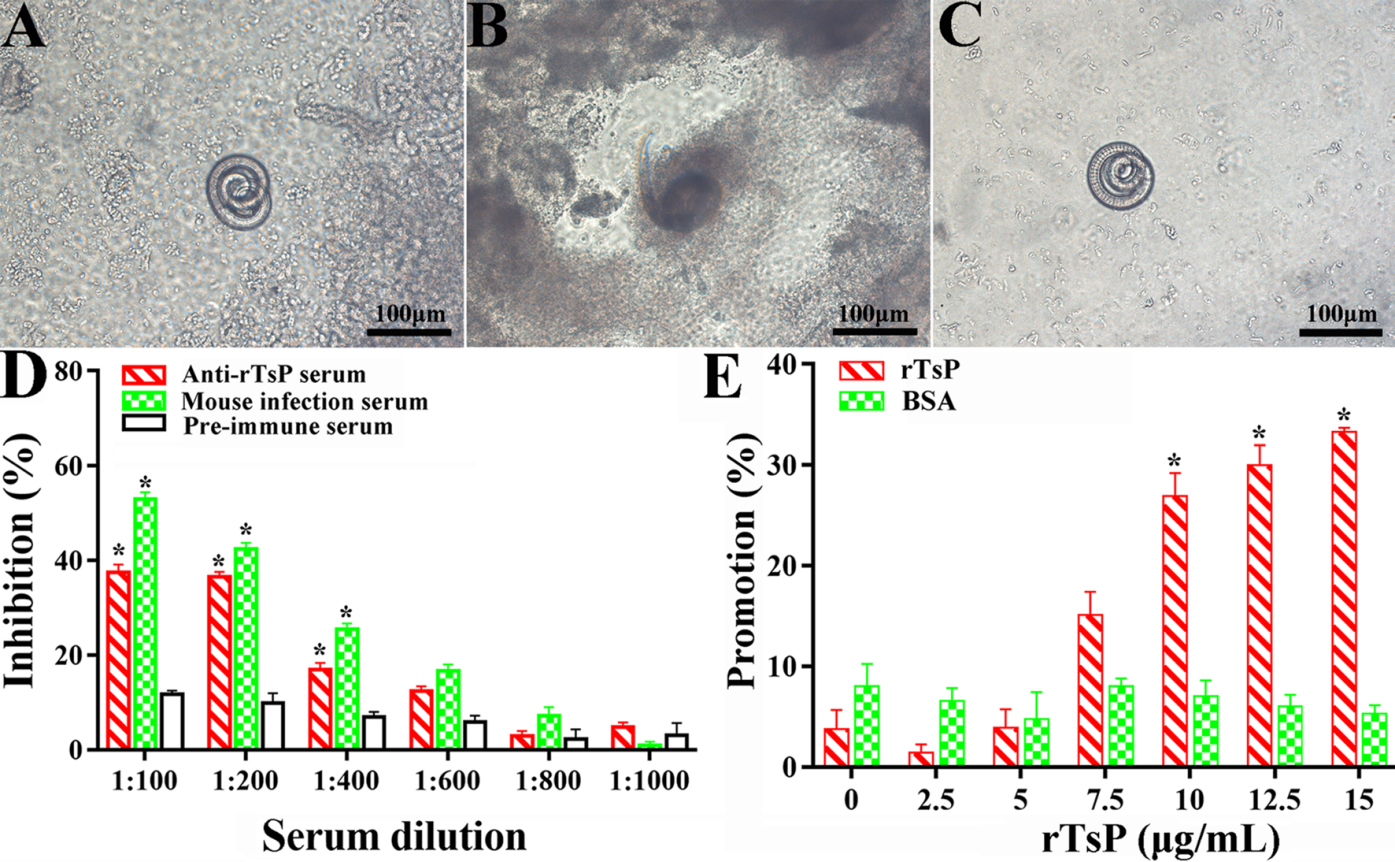
**rTsP-mediated promotion and anti-rTsP serum-mediated inhibition of the larval invasion of excised intestine.** Two hundred IIL were pre-incubated with rTsP, anti-rTsP serum, infection serum, pre-immune serum, BSA, or PBS; subsequently injected into the excised mouse gut lumen; and maintained in Tyrode’s solution at 37 °C for 2 h. The enteral segment was opened, and the enteral mucosa was compressed and observed under microscopy. **A** Non-invaded larvae showed a spiral coil-like shape within the enteral lumen. **B** Invaded larvae were inside the enteral mucosa. **C** Non-invaded larvae in the caecum were used as a negative control. **D** and **E** The inhibitory and promotive effects of the pre-incubation of larvae with anti-rTsP serum (**D**) or rTsP (**E**) on larval invasion of the enteral mucosa are shown. *P < 0.01 compared to pre-immune serum or BSA. Scale bars: 100 μm.

After they were injected and incubated for 2 h, some IIL invaded the enteral mucosa (Figure 9). When the IIL were pre-incubated with anti-rTsP serum, infection serum, pre-immune serum, rTsP, BSA or PBS, the differences in larval invasion rate among various groups were statistically significant (*P *< 0.01). Anti-rTsP serum (1:200) clearly inhibited parasite invasion of the gut epithelium compared to that upon administration of pre-immune serum (*χ*^*2*^ = 15.253, *P *< 0.0001). In contrast, 10 μg/ml rTsP clearly promoted larval invasion compared to that in the BSA group (χ^2^ = 13.665, *P* < 0.0001).

### The anti-TsP antibody response to rTsP immunization

**Figure 10:**
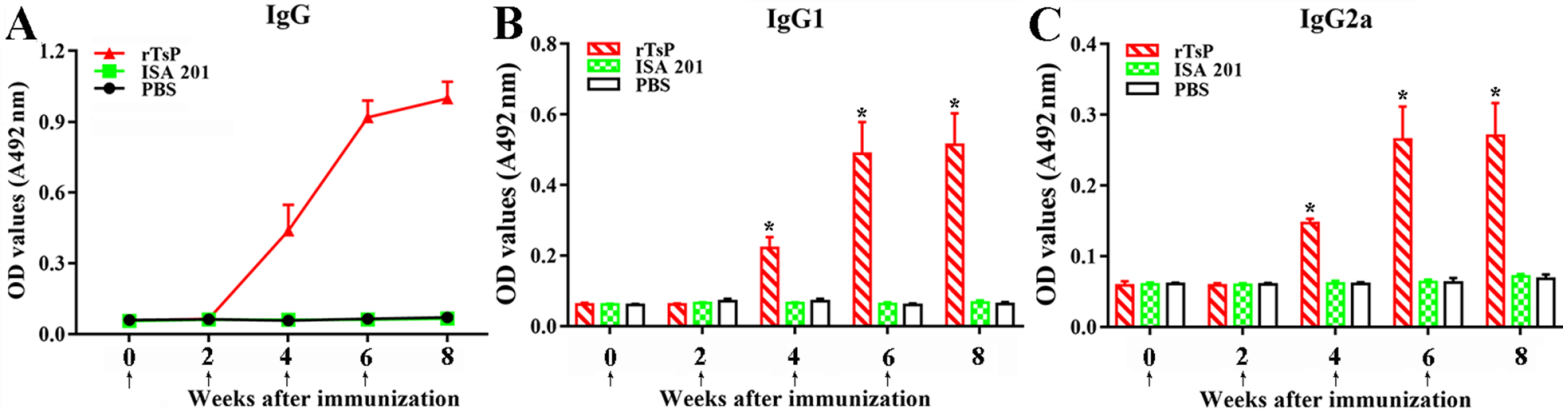
**Specific antibody responses in mice vaccinated with rTsP. A** Specific total IgG in mice immunized with rTsP, the adjuvant ISA 201 or PBS at different time points after vaccination. Specific IgG1 (**B**) and IgG2a (**C**) subclass responses against rTsP at different times after vaccination. The OD values from each group are shown as the mean ± SD of antibody levels (n = 20). The vaccination times are indicated by arrows (↑). *P < 0.0001 compared to the ISA 201 adjuvant or PBS group.

To assess the specific anti-TsP antibody response, rTsP-specific IgG and IgG1/IgG2a in the sera of vaccinated mice were measured using rTsP-ELISA. The serum anti-rTsP IgG antibody level in vaccinated mice was obviously increased following the second vaccination, and anti-rTsP IgG titres at 2 weeks after the last immunization reached 1:10^5^, indicating that rTsP was highly immunogenic. However, no mice injected with the adjuvant ISA 201 or PBS alone showed anti-rTsP IgG responses (Figure 10A). The IgG1 levels on weeks 4, 6 and 8 following immunization were distinctly higher than the IgG2a levels (*t*_4w_ = 10.451, *t*_6w_ = 9.853, *t*_8w_ = 10.841, *P* < 0.0001) (Figure 10B, C), indicating that vaccination with rTsP elicited a Th2-predominant mixed Th1/Th2 response.

### An intestinal mucosal sIgA response was elicited by rTsP immunization

**Figure 11:**
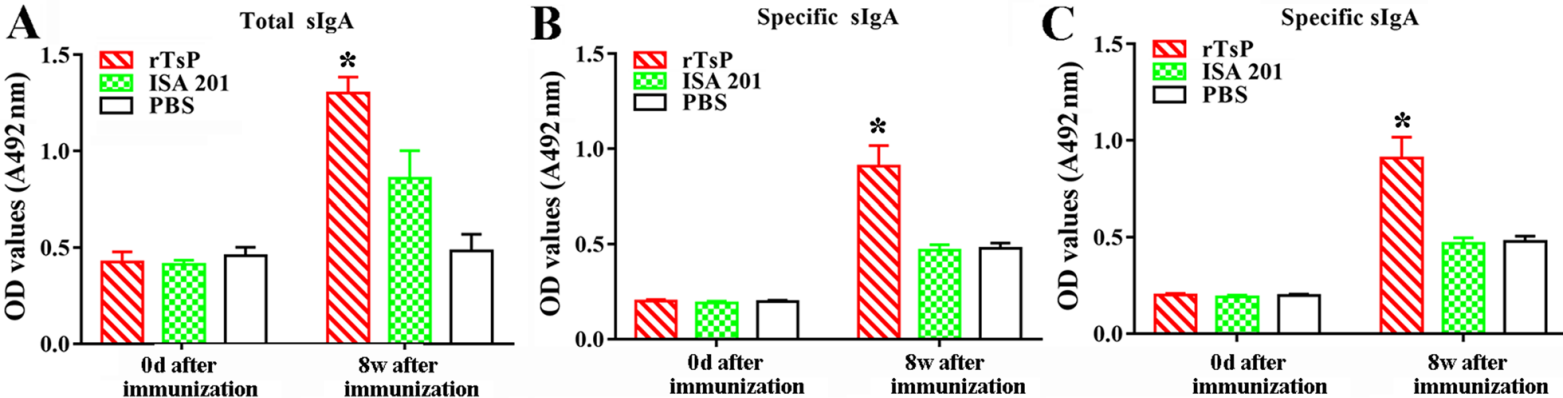
**Total sIgA (A) and TsP-specific sIgA levels in the enteral eluent of immunized mice were measured with ELISA using ML crude antigens (B) and rTsP (C).** The data are the mean OD values ± SDs of 5 mice per group. *P < 0.0001 compared to the ISA 201 adjuvant or PBS group.

To ascertain the intestinal mucosal sIgA response to rTsP immunization, total sIgA and TsP-specific sIgA levels were measured with ELISA. The results revealed that the total sIgA and TsP-specific sIgA levels among the three groups were not significantly different prior to immunization (*P *> 0.05). However, the total sIgA level at 8 weeks following immunization was clearly higher in mice immunized with rTsP than in mice administered the adjuvant ISA 201 adjuvant or PBS (*F *= 72.421, *P *< 0.0001) (Figure 11A). When crude antigens from the ML and rTsP were used, the specific sIgA level in immunized mice was significantly higher than that in the groups administered adjuvant or PBS (*F*_ML_ = 70.115, *F*_rTsP_ = 64.678; *P *< 0.0001) (Figures 11B, C). No specific intestinal sIgA response was detected in mice injected with only adjuvant or PBS. These results suggest that subcutaneous immunization with rTsP induced both a systemic antibody response and a local intestinal mucosal sIgA response.

### Cytokine responses to rTsP immunization

**Figure 12:**
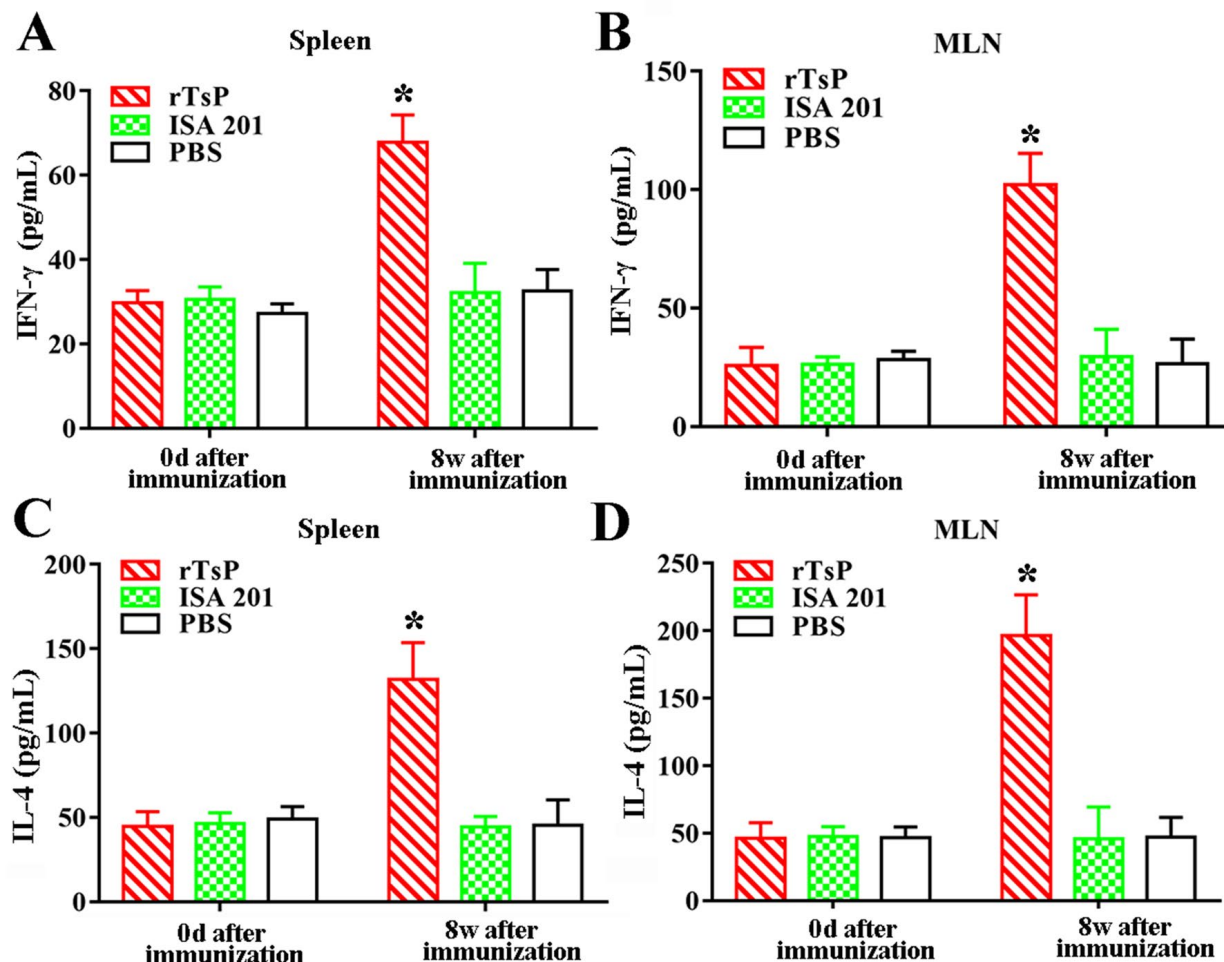
**Cytokines secreted by the spleen and mesenteric lymph nodes (MLNs) from mice immunized with rTsP.** The concentrations of two cytokines, IFN-γ (**A**, **B**) and IL-4 (**C**, **D**), were measured in the supernatant after the spleen and MLN cells were stimulated with 4 μg/mL rTsP for 72 h. The data are shown as the mean ± SD of five mice per group. *P < 0.001 compared to the ISA 201 adjuvant and PBS control groups.

To measure cytokine responses triggered by rTsP immunization, the spleens and MLN cells obtained from immunized mice were cultivated under rTsP stimulation. Supernatants were collected, and cytokine concentrations were measured by sandwich ELISA. The levels of a Th1 cytokine (IFN-γ) and a Th2 cytokine (IL-4) were distinctly elevated at 8 weeks after rTsP immunization compared to those in groups administered the adjuvant ISA 201 or PBS (*P* < 0.001) (Figure 12). Our results indicated that rTsP immunization elicited mixed Th1/Th2 responses according to the level of specific IgG subclasses and cytokines, suggesting that subcutaneous immunization with rTsP induced both systemic (spleen) and local enteral mucosal (MLN) cellular responses.

### Immune protection produced by rTsP immunization

**Figure 13:**
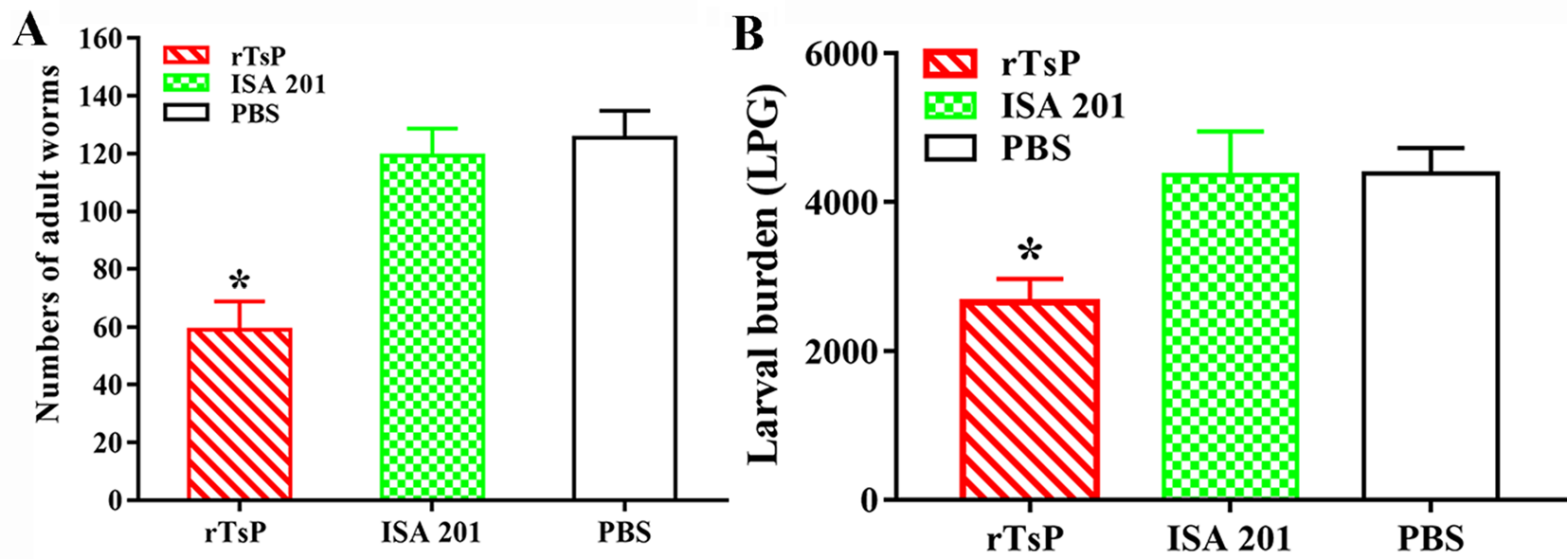
**Immune protection induced by rTsP immunization after challenge with 300** ***T. spiralis*** **larvae in a murine model. A** Intestinal AW burden. **B** Muscle larval burden (larvae per gram, LPG). The worm burdens are shown as the mean ± SD from the rTsP-immunized mice, ISA 201 adjuvant and PBS groups (n = 10). *P < 0.001 compared to the ISA 201 adjuvant and PBS groups.

Compared to mice in the PBS group, mice immunized with rTsP exhibited a 38.60% reduction in the number of intestinal AWs at 6 dpi (Figure 13A) and a 41.93% reduction in the number of ML at 30 dpi (Figure 13B) following challenge with 300 infectious *T. spiralis* larvae (*F*_AW_ = 159.895, *F*_ML_ = 132.928, *P *< 0.001). However, the mice injected with only the adjuvant ISA 201 did not show any significant reduction in the AW or ML burden (*P* > 0.05) compared to those in the PBS group. These results indicated that the immunization of mice with rTsP induced significant immune protection against *T. spiralis* larval challenge.

### Intestinal and muscle histological changes in infected mice

**Figure 14:**
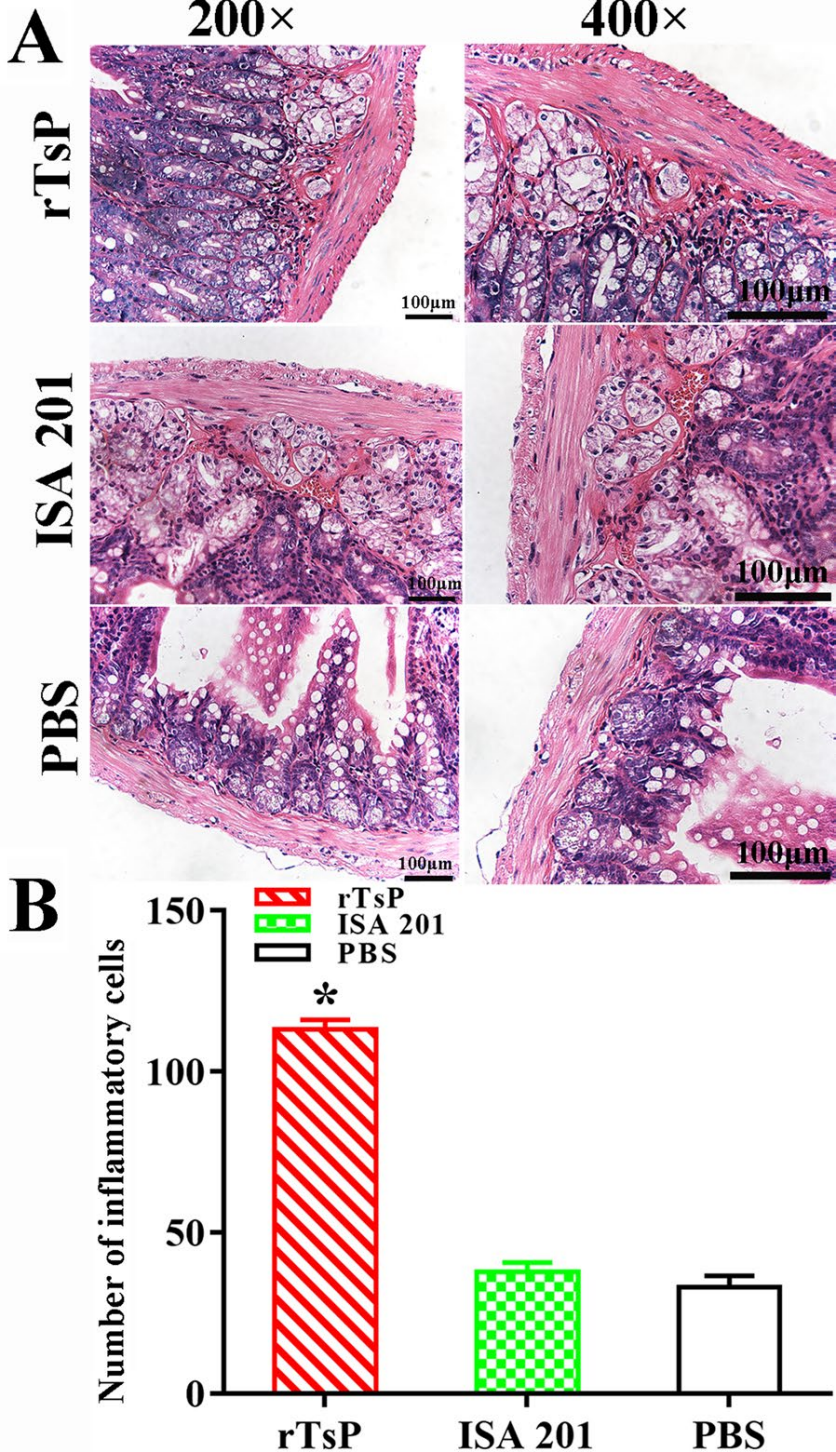
**Intestinal histopathological changes in infected mice.** The intestinal sections were stained using haematoxylin and eosin (HE) and observed under microscopy. **A** Inflammatory cell infiltration of the intestinal mucosa in three groups of infected mice. **B** Quantification of intestinal mucosal inflammatory cells in intestinal sections. *P < 0.0001 compared to the ISA 201 adjuvant and PBS groups. Scale bars: 100 μm.

**Figure 15:**
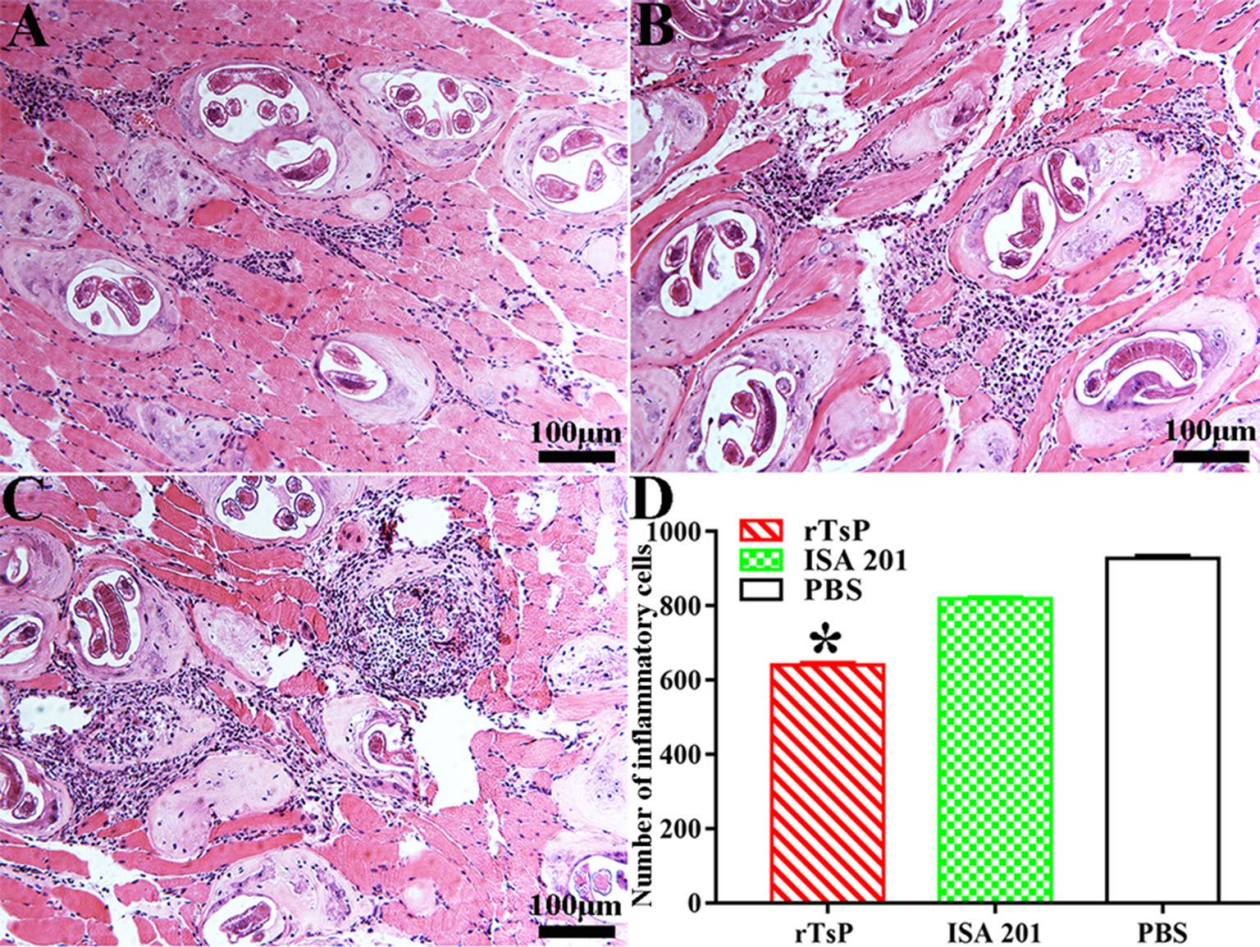
**Histopathological changes in skeletal muscles from infected mice.** The masseter muscle sections were stained using haematoxylin and eosin (HE) and examined under microscopy. **A** rTsP-immunized mice. **B** ISA 201 adjuvant-inoculated mice. **C** PBS-treated control mice. **D** Quantification of inflammatory cells around encapsulated *T. spiralis* larvae on muscle sections. *P < 0.0001 compared to the ISA 201 adjuvant and PBS groups. Scale bars: 100 μm.

Histological changes of the intestines and masseter muscles of different groups of mice were examined at 6 and 30 dpi. The number of inflammatory cells in the intestinal mucosa of rTsP-immunized mice was obviously higher than that in the mice administered adjuvant or PBS as a control (*F *= 579.160, *P* < 0.0001) (Figure 14), whereas the number of inflammatory cells around encapsulated larvae in the skeletal muscles of rTsP-immunized mice was significantly reduced compared to that in the groups administered adjuvant or PBS as a control (*F *= 1734.315, *P* < 0.0001) (Figure 15). These results suggest that rTsP immunization enhanced intestinal inflammatory cell infiltration in immunized mice, which may accelerate AW expulsion from the gut. Furthermore, rTsP immunization alleviated inflammatory infiltration and relieved *Trichinella* infection in the skeletal muscles of immunized mice.

## Discussion

In the present study, the complete sequence of the TsP gene was cloned, and the TsP gene was expressed in a prokaryotic expression system. Sequence analysis showed that TsP has an identity of 86.67, 83.40, 83.02, 82.26, 81.51, 80.90 and 80.38% with peptidases of 7 encapsulated *Trichinella* species (*T. nelsoni, T. native, T. britovi*, T9, *T. patagoniensis*, T6 and *T. murrelli*) and an identity of 74.25% with a peptidase from the non-encapsulated species *T. papuae*. A phylogenetic tree showed strongly supported the presence of a monophyletic group of the genus *Trichinella*. Bioinformatics analysis revealed that TsP contains a functional domain with trypsin-like serine protease activity containing an active site carrying the classic catalytic triad, indicating that TsP is a member of the S1 subfamily of peptidases. The rTsP protein was desaturated by dissolving in 8 M urea and renatured by gradient dialysis [38], but the enzymatic activity of rTsP was not observed by using gel zymography with a specific substrate (data not shown). The absence of serine protease enzymatic activity in rTsP might be due to the incorrect folding of rTsP in a prokaryotic expression system [53] or because TsP is secreted in an inactive (pro) form [28]. Therefore, to obtain rTsP with enzymatic activity, a eukaryotic expression system must be used. After purification, rTsP was used to generate anti-rTsP antibodies. Immunization of mice with rTsP elicited a specific anti-rTsP antibody response, and the titre of specific anti-rTsP IgG in immune serum reached 1:10^5^, indicating that rTsP has strong immunogenicity.

The results of RT-PCR showed TsP mRNA transcription in *T. spiralis* at all diverse stages examined (ML, IIL, 3- and 6-day-old AWs, and NBL). Western blot analysis revealed that several native TsP constructs with MWs of 28.7-41.7 kDa among the soluble and ES proteins of *T. spiralis* ML were identified by anti-rTsP serum. This is likely because TsP adopt have various isoforms; because this TsP protein was post-translationally modified or processed; or because TsP is a member of the *Trichinella* serine protease superfamily, the members of which possess the same antigenic epitopes [19, 38, 41]. Western blotting also indicated that TsP was expressed in ML ES proteins and that TsP is a secretory protein, suggesting that TsP is directly exposed to the host’s immune system and triggers the production of anti-TsP antibodies during *Trichinella* infection [12, 21, 39].

The IIFA results showed native TsP protein expression in *T. spiralis* at different stages (ML, IIL, AWs and NBL) and that TsP was localized dominantly at the epicuticle and of this parasite, suggesting that TsP, a primarily surface protein, might participate in *T. spiralis* intrusion and survival in the host [22]. The surface proteins of *T. spiralis* IIL are first exposed to and make direct contact with the host’s intestinal epithelium, which might mediate larval invasion of the intestinal mucosa [15, 24]. The results of IIFA and far-western blotting showed that the specific binding of rTsP with the enteral epithelium and IECs, and the results of confocal microscopy indicated that the rTsP-binding sites are primarily localized in the cytoplasm. When the larval invasion of IECs and the isolated small intestine was examined in vitro, rTsP obviously increased the larval intrusion of IECs and intestinal mucosa, and this increase was dependent on the rTsP dose, a feature that might be related to specific binding between rTsP and IECs [43, 46, 52]. Moreover, the capacity of IIL to intrude into IECs and the intestinal mucosa was notably inhibited by the administration of anti-rTsP antibodies, and this inhibitory effect dependent on the dose of anti-rTsP antibodies. The suppressive effect of anti-rTsP antibody on larval intrusion might be due to the formation of a cap-like TsP and anti-TsP antibody immune complex at the IIL anterior, which hinders the direct contact of larvae with enterocytes, thus impeding larval intrusion [62]. When *Trichinella*-infected mouse sera were used in the intrusion assay, the suppressive effect on intrusion was more pronounced than the effect of anti-rTsP serum. This is likely because antibodies against other invasion-related proteins from *T. spiralis* (e.g., cysteine proteases, aminopeptidases, glutathione S-transferase, and so on) in infected sera also exerted a suppressive effect against intrusion [45, 48]. However, it is necessary to identify which IEC proteins interact with TsP using co-immunoprecipitation, pull-down assays and a yeast two-hybrid system [63].

In the present study, to ascertain the protective immunity elicited by rTsP vaccination, the antibody and cytokine responses induced by rTsP vaccination were assessed. The results revealed that the vaccination of mice with rTsP triggered a prominent anti-TsP antibody response (high levels of IgG, IgG1/IgG2a and sIgA) and also triggered systemic (spleen) and local enteral mucosal (MLN) cellular immune responses, as shown by an obvious increase in a Th1 cytokine (IFN-γ) and Th2 cytokine (IL-4) after the spleens and MLN cells of immunized mice were stimulated using rTsP. This concomitant Th1/Th2 response plays a vital role in immune protection against *T. spiralis* infection [57, 64, 65]. Specific anti-*Trichinella* IgG participated in the rapid expulsion of worms from the intestine and NBL destruction through ADCC [42, 66, 67]. Additionally, IFN-γ plays a protective role against *T. spiralis* infection by enhancing cytotoxic killing and activating macrophages. IL-4 plays an important function in the development of resistance to *T. spiralis* infection, and when this cytokine was suppressed, the nematode survival was increased [68]. Furthermore, rTsP immunization appeared to enhance intestinal inflammatory infiltration in immunized mice, which may accelerate AW expulsion from the intestine. Muscle inflammatory infiltration in immunized mice might be alleviated after challenge because the IL-10 elicited by rTsP immunization restricts the inflammatory reaction to *T. spiralis* larvae infecting the muscle [57, 69]. Our results indicated that the immunization of mice with rTsP resulted in obvious reductions in the intestinal adult and muscle larval burdens in rTsP immunized mice. These results suggest that TsP plays an important role in the intrusion, development and survival of *T. spiralis* in hosts and that TsP is a promising candidate target molecule for vaccination against *T. spiralis* infection.

*Trichinella spiralis* is a multicellular parasitizing nematode with a complex life cycle that consists of four developmental stages (ML, IIL, AWs and NBL). Each developmental stage has stage-specific antigens. The protective immunity triggered by vaccination with an individual *T. spiralis* protein molecule is not sufficient to protect the host from *T. spiralis* challenge infection [70]. In this study, the immunization of mice with rTsP reduced ML in muscle tissues by only 41.93%; *T. spiralis* larvae were not completely eliminated in immunized animal muscles. Therefore, an effective preventive vaccine should be composed of multiple antigens that can elicit immune responses against worms at various stages of the life cycle, polyvalent vaccines against target antigens from *T. spiralis* at diverse stages of the *T. spiralis* life cycle should be developed, and their immunoprotective effects should be evaluated in future experiments [60].

In conclusion, TsP is a surface and secretory protein expressed in *T. spiralis* at diverse stages that is primarily located at the epicuticle of the parasitic nematode. rTsP has the capacity to specifically bind IECs and the intestinal epithelium, and the rTsP-binding site is localized in the IEC cytoplasm. rTsP facilitated the larval intrusion of IECs and the intestinal mucosa, whereas anti-rTsP antibodies suppressed larval intrusion; these facilitative and suppressive effects were dose-dependently related to rTsP or anti-rTsP antibodies. The immunization of mice with rTsP triggered obvious systemic and enteral local antibody and cellular immune responses, resulting in significant immune protection against *T. spiralis* challenge. These results indicate that TsP plays a major role in the intrusion, development and survival of *T. spiralis* in hosts and that TsP is a promising candidate target molecule for vaccination against *T. spiralis* infection.

## Supplementary information

**Additional file 1. Sequence alignment of the** ***Trichinella spiralis*** **peptidase gene (XP_003379348.1) with peptidase genes from other species or genotypes of the genus** ***Trichinella***. Clustal X and BOXSHADE were used to analyse the sequences, and distinct differences between peptidases from different *Trichinella* species/genotypes were observed. Black shading indicates residues identical to those in TsP, and grey shading showed conservative substitutions.

## Data Availability

Not applicable.

## Abbreviations

AWs: adult worms
DAB: 3,3′-Diaminobenzidine tetrahydrochloride
DAPI: 4′,6-Diamidino-2-phenylindole
ES: excretory/secretory
FBS: foetal bovine serum
IECs: intestinal epithelial cells
IIFA: indirect immunofluorescence assay
IIL: intestinal infective larvae
LPG: larvae per gram
ML: muscle larvae
MLN: mesenteric lymph nodes
MW: molecular weight
NBL: newborn larvae
pI: isoelectric point
SD: standard deviation
TsP: *T. spiralis* peptidase
