# Supplementary material for: Molecular cloning and characterization of a novel peptidase from Trichinella spiralis and protective immunity elicited by the peptidase in BALB/c mice
Source: Vet Res. 2020 Sep 5;51:111. doi: 10.1186/s13567-020-00838-1 (PMC7487599; doi:10.1186/s13567-020-00838-1)
Supplement: Supplementary file 1 — Additional file 1. Sequence alignment of the Trichinella spiralis peptidase gene (XP_003379348.1) with peptidase genes from other species or genotypes of the genus Trichinella. Clustal X and BOXSHADE were used to analyse the sequences, and distinct differences between peptidases from different Trichinella species/genotypes were observed. Black shading indicates residues identical to those in TsP, and grey shading showed conservative substitutions. [file 13567_2020_838_MOESM1_ESM.docx]

**
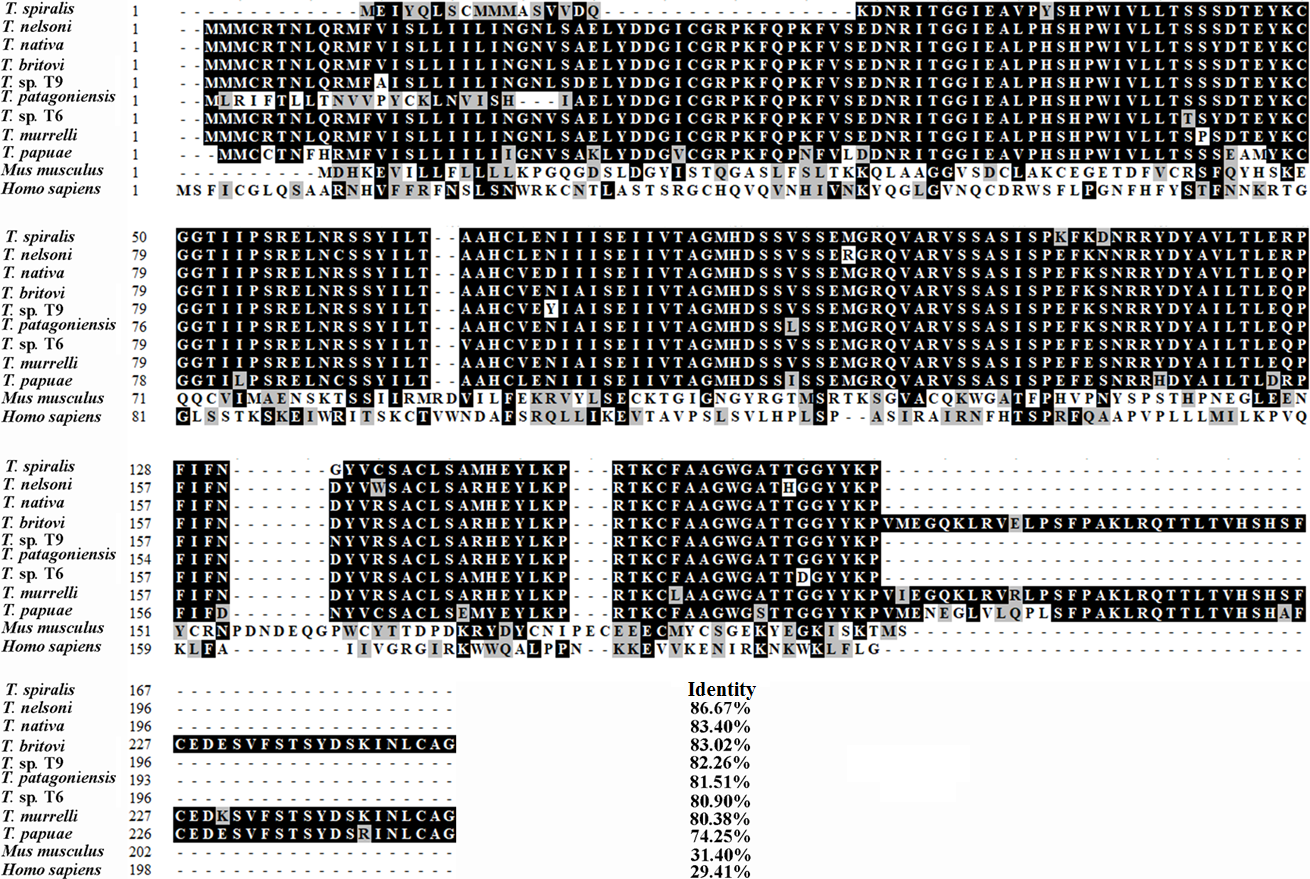
**

**Additional file 1**. **Sequence alignment of the *Trichinella spiralis* peptidase gene (XP_003379348.1) with peptidase genes from other species or genotypes of the genus *Trichinella*.** Clustal X and BOXSHADE were used to analyse the sequences, and distinct differences between peptidases from different *Trichinella* species/genotypes were observed. Black shading indicates residues identical to those in TsP, and grey shading showed conservative substitutions.
